# Phase Unwrapping Error Correction Based on Multiple Linear Regression Analysis

**DOI:** 10.3390/s23052743

**Published:** 2023-03-02

**Authors:** Zhuang Lv, Kaifeng Zhu, Xin He, Lei Zhang, Jiawei He, Zhiya Mu, Jun Wang, Xin Zhang, Ruidong Hao

**Affiliations:** 1 Changchun Institute of Optics, Fine Mechanics and Physics, Chinese Academy of Sciences, Changchun 130033, China; 2 University of Chinese Academy of Sciences, Beijing 100049, China

**Keywords:** fringe projection profilometry, phase unwrapping error, multiple linear regression analysis, error analysis

## Abstract

Fringe projection profilometry (FPP) is prone to phase unwrapping error (PUE) due to phase noise and measurement conditions. Most of the existing PUE-correction methods detect and correct PUE on a pixel-by-pixel or partitioned block basis and do not make full use of the correlation of all information in the unwrapped phase map. In this study, a new method for detecting and correcting PUE is proposed. First, according to the low rank of the unwrapped phase map, multiple linear regression analysis is used to obtain the regression plane of the unwrapped phase, and thick PUE positions are marked on the basis of the tolerance set according to the regression plane. Then, an improved median filter is used to mark random PUE positions and finally correct marked PUE. Experimental results show that the proposed method is effective and robust. In addition, this method is progressive in the treatment of highly abrupt or discontinuous regions.

## 1. Introduction

Fringe projection profilometry (FPP) [1,2,3] has been widely used in automatic detection, anthropometry, reverse design, biomedicine, virtual reality and many other fields due to its characteristics of non-contact, precision and strong robustness. Phase unwrapping is one of the most important steps in FPP [4], but during the unwrapping process in which the phase unwrapping error (PUE) is generated, the height mutation or discontinuity of the target surface or other noises when obtaining the fringe image will greatly reduce the measurement accuracy [5].

Some methods have been proposed for the detection and correction of PUE, which can be divided into pre-processing and post-processing according to different processing stages. Pre-processing is the method for PUE correction during fringe analysis. Wang [6] has proposed a Gray-code fringe order-jump error self-correction method based on shifted phase encoding to remove PUE. This method does not need to add other additional fringe patterns and can accurately and automatically correct the PUE caused by uneven reflectivity and edge ambiguity. Lu [7] has proposed a three-dimensional (3D) shape measurement method based on misaligned Gray-code light. This method pre-moves all traditional gray code patterns with a half fringe period before projection, such that the edges of the decoding order are completely interleaved with the wrapped phase. Then, each decoding order is divided into four subregions, the correct phase order constructed, and the wrapped phase unwrapped. In addition, the introduction of a virtual plane further improves measurement efficiency and accuracy. Ding has proposed a general rule for selecting specific frequencies for the time-phase unwrapping technology of specific two-frequency [8] and three-frequency [9] fringe pattern projections, which greatly improves the ability to resist PUE. Li [10] has proposed a PUE self-correction method based on a multi-frequency heterodyne method, which uses redundant measurement data to achieve self-correction without collecting additional data. However, these pre-processing methods are limited in scope for specific projection modes or need redundant projection patterns.

The post-processing method directly corrects PUE or indirectly corrects PUE correction by correcting the fringe order errors. According to different processing methods, post-processing methods can be divided into pixel-by-pixel retrieval [11,12,13], partition block recursion [14,15,16,17], global [18,19,20], and hybrid methods [21,22].

For the pixel-by-pixel retrieval method, Song [11] has used slope filtering to correct PUE. First, the line slope of the adjacent points is calculated, and the error points are then detected and corrected according to the absolute subtraction’s result between the two slopes. Ding [12] has also studied the same issue and addressed the error point by checking the monotonicity of each pair of pixels in each line and recovered it using the least square method. However, none of these methods are effective in detecting continuous error points. Zhang [13] has proposed a neighborhood retrieval correction method. First, the pixel point with the highest fringe quality is found as the original pixel and then the phase difference between the surrounding and original pixels is calculated for verification and correction. The verification and correction is repeated with the verified pixel as the new original point until all pixels have been verified. This method requires continuous verification and the replacement of original points, which is computationally intensive and time-consuming.

For the partition block recursion method, Ding [14] has divided the fringe order map according to the periodicity of the wrapped phase, then corrected the fringe order of each region by taking mode, and finally regenerated the unwrapped phase map. It takes a long time to distinguish the wrapped phase period, and the mode correction method will produce errors when the error points are dense. Then, Ma [15] used the same idea to divide each period of the wrapped phase into two parts: (−π, 0) and (0, π) with the sine function. The frequency of the fringe order occurrence of each part is counted separately during the partition correction and the fringe order with more than half the frequency considered to be the correct fringe order; otherwise, it is omitted. This method reduced the time required to distinguish the wrapped phase period, but still does not solve the problem of dense error points leading to error results. Moreover, separate statistics might result in a huge mistake, so that the order of the fringe is different in the first and second half periods. Wu [16] has improved this algorithm by taking advantage of the feature that the fringe order error often occurs in the period transformation area, dividing each period of the original wrapped phase into three segments on average. Then, the wrapped phase of each period with the middle segment is reconstructed as the reference data and the wrapped phase and fringe order of the middle segment are used to reconstruct the unwrapped phase. This method corrects the error caused by the inconsistency between the wrapped phase and fringe order, but it causes mistakes when there are dense error points in the middle segment. These three algorithms correct PUE from the period of the wrapped phase, and although the processing method is a partition block recursion method, it requires a lot of time to identify and rebuild the block. Additionally, it cannot effectively deal with the error-intensive area. In addition to using the method of wrapped phase period partitioning, Lu [17] has proposed a method for directly dividing blocks by fringe order. All regions with the same fringe order are extracted, and the larger connected regions are taken as a part of the new fringe order map. After filtering all the orders, the overall fringe order map and new unwrapped phase map are synthesized.

For the global method, Zhang [18,19,20] has used the robust principal component analytical method to correct PUE. This method relies on the sparsity of PUE and the low rank of the unwrapped phase map, but it needs to set multiple parameters in advance and different parameters need to be set for different targets. In addition to the single processing method, there are also some hybrid algorithms. Kam [21] has proposed a hybrid PUE-correction method in which, first, the median filter and standard deviation filtering are used to find the correct fringe order area. Then, the least square method is used to search the fringe order of adjacent pixels in the correct area and correct it, and the check and correction are repeated until all fringe orders are verified. Clearly, the calculation of this method is complex, and although the calculation accuracy is guaranteed, the current computing power cannot support its wide application in engineering practice. Deng [22] has divided PUE correction into two steps: coarse and fine corrections. Coarse correction uses a pre-processing method to correct the phase jump points and abrupt-change edges of the object. Fine correction uses eight-neighbor filtering to remove the remaining fringe order noises. This method fails to avoid the disadvantages of the pre-processing method and it is necessary to obtain redundant projection maps.

To sum up, it is very necessary to find a PUE-correction method with good noise-reduction performance while considering global image features. In this study, a global PUE-correction method is proposed, which takes advantage of the low rank of the unwrapped phase map and the sparsity of PUE. First, multiple linear regression analysis (MLRA) is used to obtain the regression plane of the unwrapped phase map. According to the regression plane, the tolerance is set to mark thick PUE. Then, the remaining random PUEs are marked with improved median filtering. Finally, all marked PUEs are corrected. The proposed method makes full use of the overall correlation of the unwrapped phase and has more advantages in dealing with the highly abrupt and discontinuous areas of the target surface. The rest of this study is arranged as follows. The Section 2 provides the PUE classification and theoretical aspects of the proposed method. The Section 3 exhibits the experimental results for verifying the effectiveness, robustness, and progressiveness of the proposed method. The Section 4 summarizes the study.

## 2. Theory

### 2.1. PUE and PUE Classification

**Figure 1:**
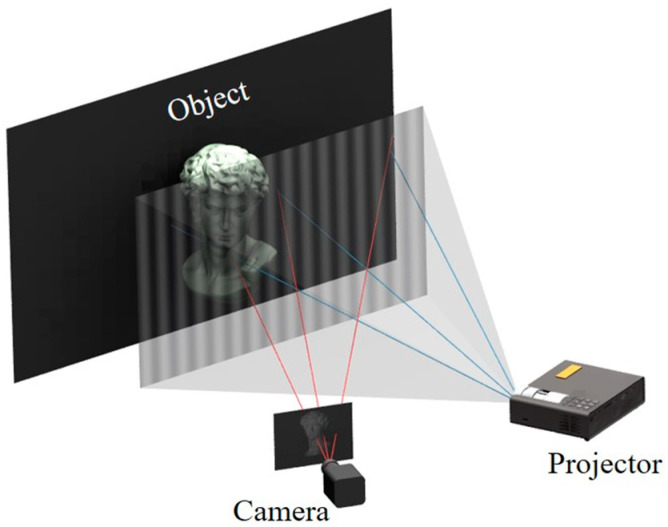
Classical fringe projection profilometry (FPP) system.

The classical FPP system shown in Figure 1 consists of a digital projector and video camera. First, a series of standard cosine fringe images are projected onto the target surface by a digital projector and then the camera collects the fringe images of the deformed surface of the object [23]. The wrapped phase is obtained through fringe analysis, the unwrapped phase is obtained after phase unwrapping [24], and finally the unwrapped phase is converted into 3D coordinates [25].

Phase unwrapping is the process of recovering the unwrapped phase from the wrapped phase. After obtaining the fringe order k(x,y) [4], the wrapped phase φ(x,y) can be unwrapped as follows:

(1) Φ(x,y)=φ(x,y)+2πk(x,y)

where Φ(x,y) is the unwrapped phase and (x,y) is the coordinate position of the pixel. However, affected by phase noise and measurement conditions, PUE will inevitably occur during phase unwrapping. According to Equation (1), PUE is divided into PUE caused by wrapped phase error and PUE caused by fringe order error.

#### 2.1.1. PUE Caused by Wrapped Phase Error

**Figure 2:**
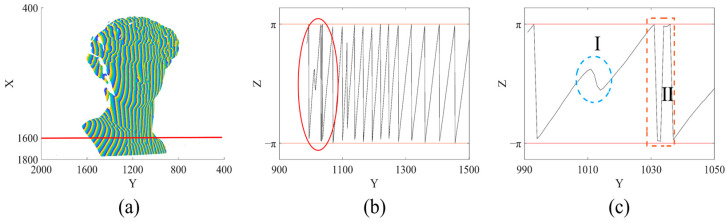
PUE caused by wrapped phase error: (**a**) Wrapped phase map; (**b**) Wrapped phase map on the section of X = 1600; (**c**) Enlarged sub-image in (**b**).

For the *N*-step phase shifting method, the wrapped phase φ(x,y) can be calculated as

(2) $$\varphi (x,y)=\arctan[\frac{\sum_{n=1}^{N}I_{n}(x,y)\sin(2n\pi /N)}{\sum_{n=1}^{N}I_{n}(x,y)\cos(2n\pi /N)}]$$

where $I_{n}(x,y)$ refers to the intensity value of arbitrary coordinate point (x,y) in the *n*th fringe pattern, and *N* is the total number of phase shifts, n=1,2,…,N. However, due to the influence of noise, errors will occur when obtaining the wrapped phase, as shown in Figure 2.

An obtained wrapped phase map is shown in Figure 2a and the wrapped phase map on the section of X = 1600 is shown in Figure 2b. There is a clear obvious wrapped phase error in the red circle, showing that the wrapped phase, which should be monotonically increasing in each cycle, has been perturbed due to noise. We enlarged the curve in the red circle in Figure 2b, as shown in Figure 2c. Wu et al. [4,26] have analyzed the types of wrapped phase errors and pointed out that the causes of wrapped phase errors are roughly divided into two types: one is a uniform and discrete error caused by noise, as shown in Region I in Figure 2c; the other is the error caused by unstable phase value at ±kπ rad (k=0,1,2,…) during the calculation of arctangent function in Equation (2), as shown in Region Ⅱ in Figure 2c. The threshold value of wrapped phase error is between $\left(-\pi ,\pi \right)$, and the PUE caused by it is small, discrete in distribution, and belongs to the category of random error.

#### 2.1.2. PUE Caused by Fringe Order Error

When the fringe order is obtained by the multifrequency heterodyne method [4], the fringe order of higher-frequency fringe pattern $k_{h}(x,y)$ can be calculated using the wrapped phase of the synthetic wavelength and the high frequency wrapped phase, expressed as

(3) $$k_{h}(x,y)=round[\frac{R\Phi_{eq}(x,y)-\varphi_{h}(x,y)}{2\pi }]$$

where function round[·] represents rounding · to its closest integer, $\Phi_{eq}(x,y)$ is the wrapped phase of the synthetic wavelength, $\varphi_{h}(x,y)$ is the high frequency wrapped phase, *R* is the fringe frequency ratio and defined as $R=f_{h}/f_{eq}$, $f_{h}$ is the high frequency wavelength frequency, and $f_{eq}$ is the synthetic wavelength frequency. The phase deviation in $\Phi_{eq}(x,y)$ is easily amplified by *R*. Once the absolute value of the amplified phase deviation exceeds 0.5π, it is likely to cause the fringe order error and then generate PUE [27].

**Figure 3:**
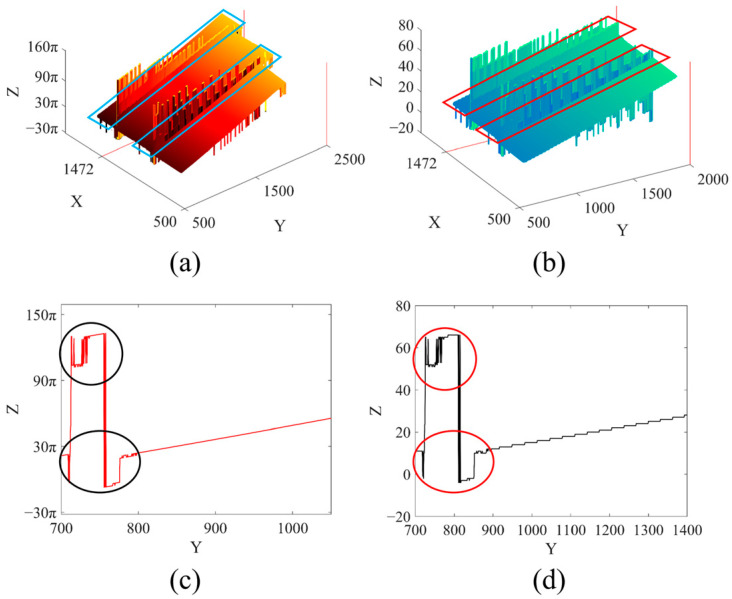
PUE caused by fringe order error: (**a**) Unwrapped phase map; (**b**) Fringe order map; (**c**) Unwrapped phase map on section of X = 1472; (**d**) Fringe order map on the section of X = 1472.

Figure 3 shows the schematic diagram of PUE caused by fringe order error. Figure 3a,b shows the unwrapped phase map and its corresponding fringe order map, respectively, and the two prominent error areas have been marked by rectangular boxes in their respective figures. The prominent errors mostly appear in the field boundary and the discontinuity or height mutation area of the measurement target surface, which is caused by the poor quality of fringe projection in this area [5,13]. This area is called here the “dense jump” area, and the PUE in this area is called the “dense jump” PUE. Comparing the error distribution and error magnitude change in the rectangular area, the two are seen to be very similar. To observe the relationship between them more intuitively, the 1472nd line of the *X*-axis of the unwrapped phase and fringe order maps are shown in Figure 3c,d, respectively. On the whole, the change trends of the two curves are consistent, error position is also consistent, and error positions are shown in the circled areas. Theoretically, the unwrapped phase should have been monotonically increased and the fringe order changed incrementally, step by step. However, the jump error (Figure 3d, circled area) greatly deviates from the expected results and the PUE (Figure 3c, circled area) also greatly distorts the measurement results. It is inferred from Equation (3) that the PUE caused by the fringe order error must be a multiple of 2π. Most PUEs caused by fringe order error, especially the dense jump PUE, belong to the category of thick error.

To sum up, the classification and analysis of PUE are carried out above. Most PUE caused by wrapped phase error can be regarded as random error and most PUE caused by fringe order error, especially dense jump error, can be regarded as thick error. Of course, the boundary between them is not absolute, but for the overall unwrapped phase, the PUE is divided into thick PUE and random PUE.

### 2.2. PUE Correction Steps of the Proposed Method

#### 2.2.1. Mark the Position of Thick PUE

For thick PUE, MLRA is used to provide a definite critical value, and any value beyond the critical range is regarded as thick PUE. The total unwrapped phase is taken as sample data and the confidence level is set to 99.99%. As the unwrapped phase has low rank, as has been proven by Zhang et al. [18], and the confidence level is large enough, the unwrapped phase is approximated as a plane, expressed as

(4) Φ(x,y)≈ax+by+d

where *a*, *b*, and *d* are the coefficients of the plane and (x,y) is the corresponding coordinate in the unwrapped phase Φ(x,y).

The above formula is then expressed in the form of a matrix

(5) Φ≈An

where, Φ is a one-dimensional sample vector composed of all Φ(x,y), A is the coefficient matrix, $A=\left(X\;Y\;1\right)$, **X**, **Y** are the coordinate vector corresponding to the sample vector Φ, and **n** is the regression coefficient vector, with $n={\left(a\;b\;d\right)}^{T}$.

To obtain the nearest regression plane

(6) $$\min{\left\|An-\Phi \right\|}^{2}$$

using the least squares method, the normal equation of Equation (6) is

(7) $$A^{T}(\Phi -An)=0$$

The general solution of this equation is

(8) $$n={\left(A^{T}A\right)}^{-1}A^{T}\Phi$$

However, because A is too large, it is extremely difficult to calculate ${\left(A^{T}A\right)}^{-1}$ in Equation (8). QR decomposition is employed to simplify this problem and decompose **A** into **Q** and **R**

(9) $$A^{m\times 3}=Q^{m\times 3}R^{3\times 3}$$

where *m* is the number of rows in **A**, **Q** is orthogonal matrix with the size of *m ×* 3, and **R** is the upper triangular matrix with the size of 3 *×* 3. Substituting Equation (9) into Equation (7) obtains

(10) $$\begin{array}{l} QRn=\Phi \\ n=R^{-1}Q\Phi \end{array}$$

After the regression coefficient is obtained, the regression matrix $\hat{\Phi }$ of Φ is constructed. The absolute value of Φ minus $\hat{\Phi }$ is

(11) $$v_{0}=\left|\Phi -\hat{\Phi }\right|$$

where $v_{0}$ is the residual error matrix.

The confidence level is 99.99%, which means that the probability of residual error falling within ±4σ (σ is the standard deviation of residual error) is 99.99%. In 15,626 measurements, the residual error $\left|\nu \right|>4\sigma$ only once. If the residual error is greater than 4σ, it is identified as thick PUE and marked. Here, ±4σ is taken as the identification critical range of thick PUE, expressed as

(12) $$\Phi_{0}(x,y)=\left\{\begin{array}{c} \Phi (x,y)\\ nan \end{array}\begin{array}{c} \left|v_{0}(x,y)\right|\leq 4\sigma \\ \left|v_{0}(x,y)\right|>4\sigma \end{array}\right.$$

where $v_{0}(x,y)$ is the value at the coordinate (x,y) in $v_{0}$, the new unwrapped phase matrix obtained after detection is $\Phi_{0}$, and $\Phi_{0}(x,y)$ is the value at the coordinate (x,y) in $\Phi_{0}$.

#### 2.2.2. Mark the Position of Random PUE

After MLRA, the thick PUE in the unwrapped phase is marked, but there are still random PUEs that have not been processed. Using the median filter to mark random PUEs and the unwrapped phase matrix $\Phi_{0}$ after the median filter is $\Phi_{1}$, the absolute value of $\Phi_{1}$ minus $\Phi_{0}$ is

(13) $$v_{1}=\left|\Phi_{1}-\Phi_{0}\right|$$

where $v_{1}$ is the residual error matrix.

To ensure the accuracy of the marking results and avoid judging the correct values as random PUEs, only the position with a jump of more than 2π is marked and the rest of the original measurement data are maintained, expressed as

(14) $$\Phi_{2}(x,y)=\left\{\begin{array}{c} \Phi_{1}(x,y)\\ nan \end{array}\begin{array}{c} \left|v_{1}(x,y)\right|<2\pi \\ \left|v_{1}(x,y)\right|\geq 2\pi \end{array}\right.$$

where $v_{1}(x,y)$ is the value at the coordinate (x,y) in $v_{1}$, $\Phi_{1}(x,y)$ is the value at the coordinate (x,y) in $\Phi_{1}$. The new unwrapped phase matrix obtained after locating is $\Phi_{2}$, and $\Phi_{2}(x,y)$ is the value at the coordinate (x,y) in $\Phi_{2}$.

#### 2.2.3. Correct the Marked PUE

At this point, random PUE has been effectively processed, but marked PUE has not been corrected. First, we calculate the unwrapped phase difference between the marked coordinates $\left(x_{0},y_{0}\right)$ and their adjacent coordinates

(15) $$\Delta \Phi (x_{0},y_{0})=\Phi (x_{0},y_{0}+1)-\Phi (x_{0},y_{0})$$

Then, the marked PUE are corrected according to the formula

(16) $$\Phi_{3}(x_{0},y_{0})=\Phi (x_{0},y_{0})-2\pi (round[\frac{\Delta \Phi (x_{0},y_{0})}{2\pi }])$$

where function round[·] represents rounding · to its closest integer. The unwrapped phase map obtained after correction using Equation (16) is $\Phi_{3}$ and $\Phi_{3}(x_{0},y_{0})$ is the value at the coordinate (x,y) in $\Phi_{3}$.

To sum up, the detailed steps of the proposed method are:

(1) Use Zhu’s method [28] to obtain the unwrapped phase matrix Φ;
(2) Use MLRA to construct the regression matrix $\hat{\Phi }$ of Φ;
(3) Mark the position of thick PUE in Φ according to Equation (12);
(4) Mark the position of random PUE in Φ according to Equation (14);
(5) Correct the marked PUE according to Equation (16).

At present, most of the existing algorithms are aimed at checking and correcting PUE caused by fringe order error and there has been little discussion regarding PUE caused by the wrapped phase. In addition, these algorithms have poor processing effects on dense jump PUE. Based on global information of unwrapped phase maps, the proposed method deals with both PUE caused by wrapped phase error and PUE caused by fringe order error. In addition, because the proposed method checks and corrects PUE according to the correlation of global information and is not restricted by the correlation of local PUE, the proposed method is more advanced in the recognition of dense jump PUE.

## 3. Experiments

The experimental system is mainly composed of a DLP projector (Light-Crafter4500, TI, Dallas, TX, USA) with a resolution of 912 × 1140 and a CMOS black-and-white camera (MV-CH050-10UM, Hikvision, City of Industry, CA, USA) with a resolution of 2048 × 2448. In this study, we obtained the wrapped phase using a four-step phase shift and unwrapped the phases using a three-frequency multi-wavelength phase unwrapping method [28].

### 3.1. Effectiveness Verification

**Figure 4:**
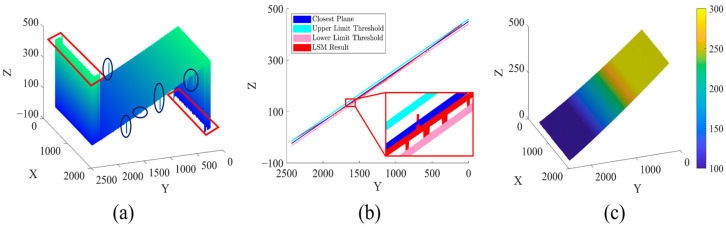
PUE-correction process of the proposed method: (**a**) Original unwrapped phase map obtained by Zhu’s method; (**b**) Unwrapped phase map after marking position of thick PUE; (**c**) Unwrapped phase map corrected by the proposed method.

In experiment 1, the procedure and effect of the proposed method in Section 2 are elaborated by correcting the PUE of a simple plane to verify the effectiveness of the proposed method. The experimental results are shown in Figure 4.

The experimental results of the unwrapped phase map of the simple plane, obtained after projection and phase unwrapping with Zhu’s method [28], are shown in Figure 4a. Due to poor fringe quality, a large number of dense PUEs are generated at the edge of the field of view (Figure 4a, red rectangular area). These dense PUEs are quite different from the actual measurement results and belong to the category of thick error. In addition, there are also some smaller PUEs (Figure 4a, blue circular area). Some of these errors deviate from the measurement results as thick errors, while others are relatively small as random errors. The results obtained after MLRA are shown in Figure 4b. The blue plane is the regression plane $\hat{\Phi }$ (Figure 4b, enlarged area, blue plane), the light blue and pink planes are the upper and lower limit threshold planes $\hat{\Phi }\pm 4\sigma$, and the red is the unwrapped phase map $\Phi_{0}$ after marking the position of thick PUE. There are some small random errors observed to be left within the upper and lower limit thresholds plane in the red unwrapped phase map. The new unwrapped phase map $\Phi_{3}$, shown in Figure 4c, is obtained after marking the positions of random PUE and correcting marked PUE. The surface of the unwrapped phase map is smooth and continuous, with both random and thick PUE removed. The experimental results show the proposed method to be effective for PUE correction.

### 3.2. Robustness Test

**Figure 5:**
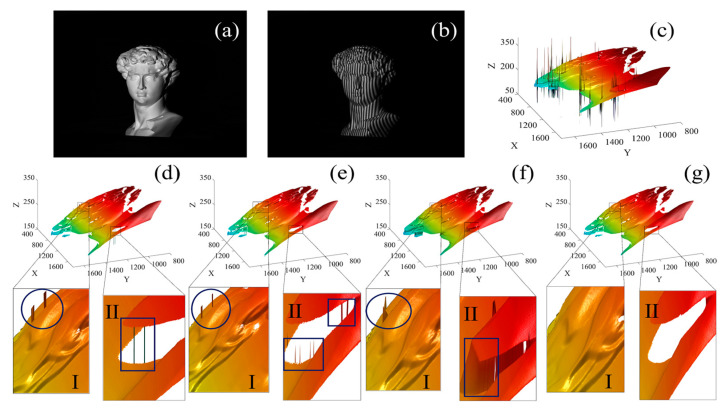
David statue model and its PUE-correction results using the comparison methods and the proposed method, respectively: (**a**) Original image; (**b**) Projected fringe pattern; (**c**) Unwrapped phase map obtained by Zhu’s method; (**d**) The unwrapped phase map corrected by Ding’s method; (**e**) The unwrapped phase map corrected by Song’s method; (**f**) The unwrapped phase map corrected by Lu’s method; (**g**) The unwrapped phase map corrected by the proposed method.

**Figure 6:**
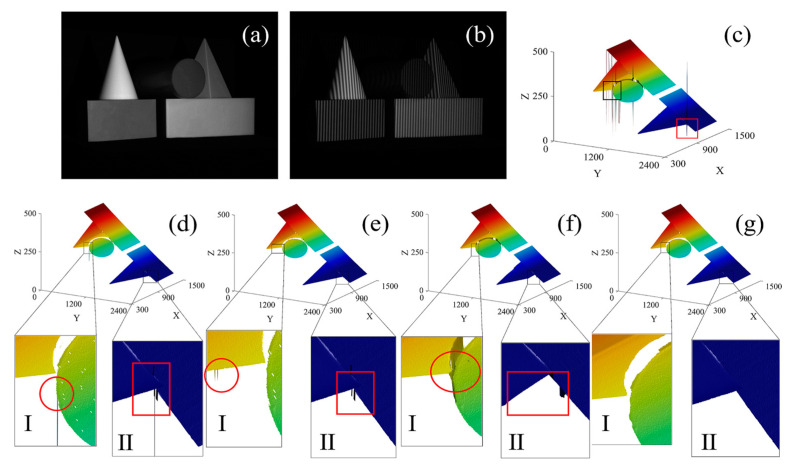
Geometry combination model and its PUE-correction results using the comparison methods and the proposed method, respectively: (**a**) Original image; (**b**) Projected fringe pattern; (**c**) Unwrapped phase map obtained by Zhu’s method; (**d**) The unwrapped phase map corrected by Ding’s method; (**e**) The unwrapped phase map corrected by Song’s method; (**f**) The unwrapped phase map corrected by Lu’s method; (**g**) The unwrapped phase map corrected by the proposed method.

To evaluate the robustness of the proposed method in complex measurement scenarios, PUE correction is carried out on the David statue model with a complex surface (Figure 5a) and the model composed of multiple geometry with a smooth surface (Figure 6a). Except for the proposed method, Ding’s monotonicity method [12], Song’s slope filtering method [11], and Lu’s decomposition and re-composition method [17] are used to carry out PUE correction for the same unwrapped phase map.

In experiment 2, the David statue model with a complex surface is taken as the measurement target. The original image and projected fringe pattern are shown in Figure 5a,b, respectively. The unwrapped phase map obtained by Zhu’s [28] method is shown in Figure 5c. Unwrapped phase maps obtained after PUE correction by Ding’s monotonicity method, Song’s slope filtering method, Lu’s decomposition and re-composition method and the proposed method are graphed (Figure 5d–g, respectively). The hair part of the David statue model has a complex structure with frequent ups and downs, which is used as evaluation Region I, and the neck of the David statue model has a large height difference, which is used as evaluation Region II. We provide the local enlarged view of Region I and Region II of each method.

Compared with the local enlarged Region I in Figure 5d,e, the residual PUE of Ding’s method and Song’s method are seen to be similar. This is caused by the same limitation of the two methods in dealing with the dense error areas. When continuous error occurs, these two methods will become ineffective. Moreover, the processing effect of these two methods in Region II is also not ideal. Ding’s method has large residual thick error. Song’s method is not accurate in the area with large height difference, which is caused by the discriminant tolerance set by the slope filtering method. There are clear residual random errors in Figure 5f Region I. The highly discontinuous region of Region II is not distinguished and there is clear distortion in the highly discontinuous edge, which is caused by the omission of correct data in the decomposition and re-composition process. In Figure 5g Region I, the PUE correction in the complex undulation area is precise, without abrupt changes. In Region II, the height abrupt boundary is accurately distinguished, and the edge is smooth without error. Compared with other methods, the proposed method has the better processing effect.

In experiment 3, PUE correction is performed on the model composed of multiple geometry with a smooth surface, and the experimental results are shown in Figure 6.

Figure 6a,b shows the original image and projected fringe pattern of this model, respectively. The unwrapped phase map obtained by Zhu’s [28] method shows that a large number of PUEs appear at the intersection of the cylinder and pyramid and at the junction of the cone and cuboid (Figure 6c, rectangular box). These two positions are taken as the focus. The intersection of the cylinder and pyramid is Region I, and the junction of the cone and cuboid is Region II. In Figure 6d, the result of Ding’s monotonicity method shows large residual PUE in the two regions, which is caused by the limitations of the monotonicity method in processing dense errors. The residual error at Region I is due to the fact that error points jump downward at the beginning of retrieval, which leads to the failure of the monotonicity method. The residual error at Region II is due to the density of error points. When the error points occur continuously, the monotone method does not distinguish error points from correct points. In Figure 6e, the results of Song’s slope filtering method shows some small errors left in Region I, which is because the errors are not within the tolerance range. However, if the tolerance is set too low, some correct points will also be recognized as error points. The reason for residual errors in Region II is the same as that for Ding’s method, being caused by the failure to correctly identify error points when they occurred continuously. In Figure 6f, the results of Lu’s decomposition and re-composition method show that Region I fails to distinguish the boundary between the pyramid and cylinder, and the arc edge of the cylinder is distorted. The distortion of Region II is clearer and a region is missing from the hypotenuse of the cone. This is because Lu’s method first decomposes the unwrapped phase map according to the fringe order and then recomposes this map using blocks with larger connected areas. A portion of the correct data is lost when selecting new blocks, resulting in inaccurate correction results. In Figure 6g, the results of the proposed method show that Region I accurately distinguishes the boundary between the pyramid and cylinder. Region II shows accurate recognition of two objects at the junction of the cone and cuboid, and the PUE in the unwrapped phase map is effectively corrected. Compared with the processing results of other methods, the proposed method has the better processing results.

The results of experiment 1 and experiment 2 show that the proposed method can effectively deal with the surface of the measured target, no matter if it is simple or complex. Combined with the results of experiments 1 and 3, it is seen that whether it is a single measurement target or multiple measurement targets, and whether the measurement target surface is continuous or discontinuous, the proposed method can effectively deal with it. Combining the results of experiments 1, 2, and 3, the proposed method is seen to be applicable to a variety of complex measurement scenarios, which confirms the strong robustness of the proposed method. This strong robustness is caused by the use of the correlation of global information in the proposed method. It is thus easier to identify dense jump PUE, which we will discuss in detail in Section 3.3.

### 3.3. Ability to Handle Dense Jump PUE

**Figure 7:**
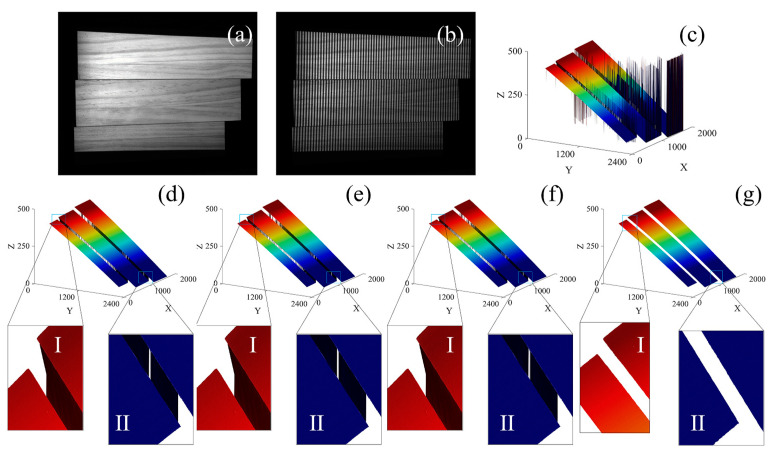
The step model and its PUE-correction results using the comparison methods and proposed method: (**a**) Original image; (**b**) Projected fringe pattern; (**c**) Unwrapped phase map obtained by Zhu’s method; (**d**) The unwrapped phase map corrected by Ding’s method; (**e**) The unwrapped phase map corrected by Song’s method; (**f**) The unwrapped phase map corrected by Lu’s method; (**g**) The unwrapped phase map corrected by the proposed method.

In this study, PUE is divided into thick PUE and random PUE. Most of the thick PUE occurs at the height mutation or discontinuity of the target surface; that is, the dense jump area. The proposed method takes the overall characteristics of the unwrapped phase as the basis for PUE correction and has more advantages in dealing with these thick PUEs. To verify this, the step model with high mutation is taken as the measurement target. Design experiments are performed to maximize thick PUEs and compared with Ding’s monotonicity, Song’s slope filtering, and Lu’s decomposition and re-composition methods. The experimental results are shown in Figure 7.

Figure 7a,b shows the original image and projected fringe pattern of the step model, respectively. The whole step model is almost full of the field of view, and from top to bottom are steps 1, 2, and 3. The height difference of each step is very large and there is little shadow at the junction of the steps. The unwrapped phase map, shown in Figure 7c, is obtained by Zhu’s method [28]. There is a large number of thick PUEs seen at the junction of each step and the boundary of the field of view. Focusing on the processing results of the boundary of the field of view and step junctions, the junction of steps 2 and 3 of the left field boundary are selected as Region I and the junction of steps 1 and 2 of the right field boundary are taken as Region II.

In Figure 7d, the correction results of Ding’s method show that, on the whole, a large number of thick PUEs remain at the step junction and edge of the boundary of the field of view. Moreover, there is a large number of residual random PUEs in both Region I and Region II. The monotonic method has poor effect in this measurement scenario. In Figure 7e, the correction results of Song’s method show that, compared with Ding’s method, the overall effect makes some progress and eliminates some thick errors, but there are no clear improvements in the processing of random PUE in Region I and Region II. This is determined by the consistency of the retrieval methods of the two methods. Both of them analyze the characteristics of a single column of pixels and corrected each column of pixels as a separate sample. This one-sidedness leads to the failure of the two methods in dense jump areas. In Figure 7f, the correction results of Lu’s method show that the PUEs have been effectively processed, which is determined by the principle of decomposition and re-composition. This method eliminates data with a small area in the unconnected area, but the random errors in Region I and Region II have not been effectively dealt with. Recognition failed due to the large area of the connected areas of random errors. In Figure 7g, the correction results of the proposed method show that, on the whole, all thick errors have been effectively dealt with, and from the local enlarged view, random PUE is also corrected. The step boundary layer is clear and identification is accurate, with field boundaries clearly divided. Compared with other methods, the proposed method exhibits better processing effects.

In comparison to other algorithms, the advantages of the proposed method in dealing with PUE, especially dense jump PUE, are verified. In fact, this is determined by the progressiveness of the proposed method in retrieving PUEs using overall data characteristics. Ding’s monotonicity method and Song’s slope filtering method use a pixel-by-pixel retrieval method, while the decomposition and re-composition method uses a partition block retrieval method. These methods only use the local data characteristics of the unwrapped phase map for PUE correction and the retrieval accuracy is inferior to the proposed method using global retrieval. PUE correction using the global search method can better distinguish the PUE that is inconsistent with the overall characteristics.

## 4. Conclusions

To solve the problem of PUE correction, this study proposes the MLRA method to identify and correct PUE according to the overall characteristics of the unwrapped phase map. First, PUE is divided into thick PUE and random PUE. Then, the MLRA method is used to obtain the optimal regression plane and the tolerance is set on the basis of the optimal regression plane to mark the positions of thick PUE. For random PUE, improved median filtering is used to mark the positions of random PUE. Finally, PUE at the mark position is corrected. As the proposed method uses global characteristics as the basis for error retrieval and recognition, this method is more sensitive for PUE recognition, especially dense jump PUE. Experiments show that the proposed method is highly robust and suitable for various complex measurement scenes and possesses more progressiveness for processing dense jump areas.

## Data Availability

Not applicable.
